# Astrocyte pathology in a human neural stem cell model of frontotemporal dementia caused by mutant TAU protein

**DOI:** 10.1038/srep42991

**Published:** 2017-03-03

**Authors:** Anna-Lena Hallmann, Marcos J. Araúzo-Bravo, Lampros Mavrommatis, Marc Ehrlich, Albrecht Röpke, Johannes Brockhaus, Markus Missler, Jared Sterneckert, Hans R. Schöler, Tanja Kuhlmann, Holm Zaehres, Gunnar Hargus

**Affiliations:** 1 Max Planck Institute for Molecular Biomedicine, Department of Cell and Developmental Biology, 48149 Münster, Germany; 2 Institute of Neuropathology, University Hospital Münster, 48149 Münster, Germany; 3 Group of Computational Biology and Systems Biomedicine, Biodonostia Health Research Institute, 20014 San Sebastián, Spain; 4 IKERBASQUE, Basque Foundation for Science, 48011 Bilbao, Spain; 5 Ruhr-University Bochum, Medical Faculty, Department of Anatomy and Molecular Embryology, 44801 Bochum, Germany; 6 Institute for Human Genetics, University of Münster, 48149 Münster, Germany; 7 Institute of Anatomy and Molecular Neurobiology, Westfälische-Wilhelms University, 48149 Münster, Germany; 8 DFG Research Center for Regenerative Therapies, Technische Universität Dresden, 01307 Dresden, Germany; 9 Westphalian Wilhelms University Münster; Medical Faculty, 48149 Münster, Germany; 10 Department of Pathology and Cell Biology, Columbia University Medical Center, New York, New York 10032, USA

## Abstract

Astroglial pathology is seen in various neurodegenerative diseases including frontotemporal dementia (FTD), which can be caused by mutations in the gene encoding the microtubule-associated protein TAU (*MAPT*). Here, we applied a stem cell model of FTD to examine if FTD astrocytes carry an intrinsic propensity to degeneration and to determine if they can induce non-cell-autonomous effects in neighboring neurons. We utilized CRISPR/Cas9 genome editing in human induced pluripotent stem (iPS) cell-derived neural progenitor cells (NPCs) to repair the FTD-associated N279K *MAPT* mutation. While astrocytic differentiation was not impaired in FTD NPCs derived from one patient carrying the N279K *MAPT* mutation, FTD astrocytes appeared larger, expressed increased levels of *4R-TAU* isoforms, demonstrated increased vulnerability to oxidative stress and elevated protein ubiquitination and exhibited disease-associated changes in transcriptome profiles when compared to astrocytes derived from one control individual and to the isogenic control. Interestingly, co-culture experiments with FTD astrocytes revealed increased oxidative stress and robust changes in whole genome expression in previously healthy neurons. Our study highlights the utility of iPS cell-derived NPCs to elucidate the role of astrocytes in the pathogenesis of FTD.

Frontotemporal dementia (FTD) is a group of neurodegenerative diseases characterized by profound degeneration of the frontal and temporal lobes leading to early-onset dementia with impairment of language, behavior and cognition^1^. FTD is the second most common dementia after Alzheimer’s disease (AD) and can be caused by mutations in *MAPT* on chromosome 17 encoding the microtubule-associated protein TAU, which stabilizes microtubules, promotes neural outgrowth and protects DNA from heat damage and oxidative stress^2^,^3^. Patients with mutant *MAPT-*associated FTD (also known as *FTD and Parkinsonism linked to chromosome 17* or FTDP-17) suffer from additional degeneration of brain stem areas including the substantia nigra and demonstrate excessive accumulation of phosphorylated TAU protein (p-TAU) in neurons and also in astrocytes, which undergo pronounced morphological changes in affected brain areas^4^. Some patients, including those with the N279K mutation in *MAPT*, also show pathologically increased levels of 4R-TAU isoforms within the brain parenchyma representing splice variants of *MAPT* containing exon 10^4^.

Despite well-documented histomorphologic changes of astrocytes in FTD, it is currently not known if astrocytes play an active role in the pathogenesis of FTD. In fact, it is unknown if astrocytes carry intrinsic detrimental programs leading to cellular degeneration and if astrocytes have the ability to influence neighboring neurons to trigger neuronal degeneration as previously described in mouse and cell models of amyotrophic lateral sclerosis (ALS)^5^,^6^. These questions have important implications for understanding and potentially interfering with disease development in FTD, given that astrocytes carry important functions in the normal brain such as supporting neurogenesis and synaptogenesis, providing trophic support to neurons and protecting neurons from oxidative stress^7^. To address this topic, we used induced pluripotent stem (iPS) cell-derived neural progenitor cells (NPCs) from one FTD patient carrying the N279K *MAPT* mutation, a mutant NPC-derived isogenic control and NPCs from one healthy control individual to establish a human stem cell model of astrocyte pathology in FTD.

## Results and Discussion

### Derivation and characterization of FTD and control NPCs

**Figure 1:**
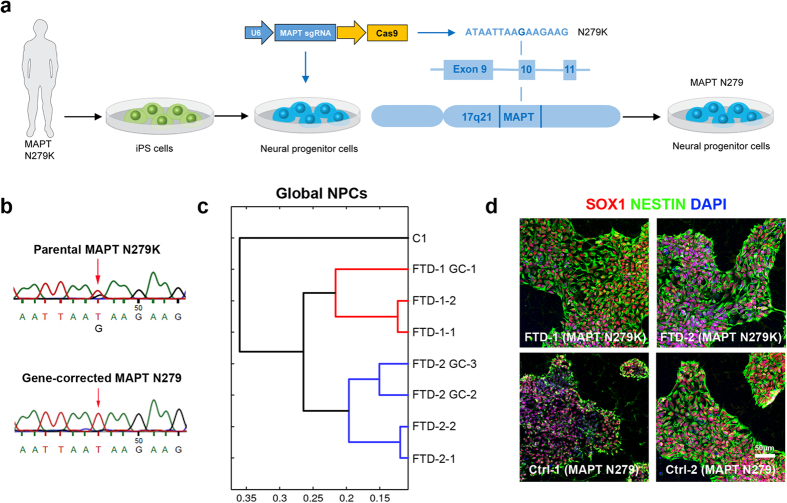
Generation of human FTD and control neural progenitor cells. **(a**) Schematic drawing depicting the genetic correction of the FTD-causing N279K mutation in exon 10 of the *MAPT* gene in human induced pluripotent stem (iPS) cell-derived neural progenitor cells (NPCs) using CRISPR/Cas9 technology. **(b)** DNA sequencing electropherograms from genomic DNA of FTD NPCs carrying the heterozygous N279K *MAPT* mutation and of CRISPR/Cas9-gene-corrected N279 *MAPT* Ctrl NPCs (clone FTD-1 GC-1). **(c)** Hierarchical cluster analysis of whole genome expression profiles in FTD and Ctrl NPCs. **(d)** Immunostaining of FTD and Ctrl NPCs for SOX1 (red) and NESTIN (green). Nuclei were counterstained with DAPI (blue). Scale bar = 50 μm.

NPCs were generated from human iPS cells carrying the N279K *MAPT* mutation (FTD NPCs) and were differentiated in parallel with human iPS cells from a healthy control (Ctrl) individual carrying wildtype *MAPT* (C1 NPCs). In addition, we performed CRISPR/Cas9 genome editing in FTD NPCs to correct the N279K mutation in exon 10 of the *MAPT* gene (Fig. 1a). We used dual expression of single guide RNAs (sgRNAs) and Cas9 from a single vector, a single-stranded DNA oligonucleotide (ssODN) providing the wildtype sequence as well as short-term puromycin selection to establish NPC clones carrying the gene-corrected *MAPT* locus (*MAPT* N279). We were able to expand rescued NPC clones with a frequency of six out of 25 clones according to diagnostic genomic restriction analysis (Fig. S1a). From these six clones, one was karyotypically abnormal, another showed aberrant gene expression and a third could not be propagated further. Genomic sequencing verified the genetic correction in exon 10 of *MAPT* in Ctrl NPCs (Fig. 1b). Global transcriptome analysis revealed high similarity between gene-corrected clones (FTD-1 GC-1, FTD-2 GC-2, FTD-2 GC-3) and their parental NPC lines (FTD-1, FTD-2) as visualized in the heat map expression profile analysis (Fig. S1c). Hierarchical clustering analysis demonstrated that gene-corrected lines clustered closest with their parental lines and separated from the unrelated, non-isogenic healthy C1 Ctrl NPC line (Fig. 1c). All NPC lines could be efficiently expanded and showed characteristic, strong expression of the NPC markers SOX1 and NESTIN (Fig. 1d).

### FTD NPCs demonstrate efficient differentiation into neurons and astrocytes *in vitro*

We first applied a well-established differentiation protocol to differentiate FTD (FTD-1 and FTD-2) and Ctrl (C1 and FTD-1 GC-1, henceforth termed Ctrl-1 and Ctrl-2) NPCs into MAP2-expressing neurons (Fig. S1d)^8^,^9^,^10^. This approach revealed efficient neuronal differentiation in both groups and demonstrated that neuronal differentiation per se was not impaired in FTD NPCs, as similarly seen in a previous study on N279K *MAPT* mutant neurons^10^.

**Figure 2:**
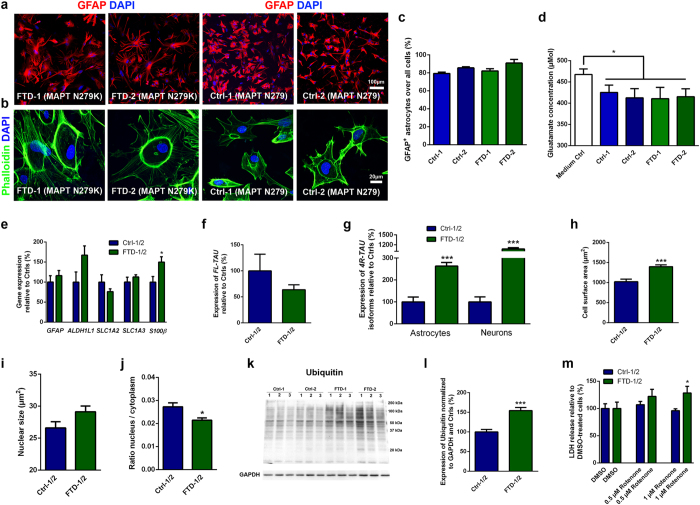
Generation of astrocytes from control and FTD NPCs and characterization of disease phenotypes in differentiated FTD astrocytes. **(a)** Immunostaining of FTD and Ctrl astrocytes for GFAP (red). Nuclei were counterstained with DAPI (blue). Scale bar = 100 μm. **(b)** Staining of FTD and Ctrl astrocytes for Phalloidin (green). Nuclei were counterstained with DAPI (blue). Scale bar = 20 μm. **(c)** Quantification of astrocytic differentiation of FTD and Ctrl NPCs. Data are represented as mean of replicates from three independent differentiation experiments (n = 3 per line) + SEM. **(d)** Quantification of glutamate uptake in astrocytes. Data are represented as mean of replicates from three independent differentiation experiments (n = 3 per line) + SEM. **(e)** qRT-PCR expression analysis of astrocyte marker genes in FTD and Ctrl astrocytes. **(f)** Quantification of full-length *TAU (FL-TAU*) expression in FTD and Ctrl astrocytes via qRT-PCR. **(g)** qRT-PCR expression analysis of *4R-TAU* isoforms in FTD and Ctrl astrocytes and neurons. **(h–j)** Quantification of the cell size (**h**), nuclear size (**i**) and nucleus/cytoplasm ratio (j) of FTD and Ctrl astrocytes. **(k)** Western blot expression analysis of ubiquitin in FTD and Ctrl astrocytes. GAPDH was used as loading control. Independent replicates are shown for each line. Images have been cropped using Photoshop software. Full-length blots are presented in Fig. S5a,b. **(l)** Quantification of ubiquitin in FTD and Ctrl astrocytes. Data in panels e–j and l are represented as mean of replicates from three independent differentiation experiments per line (n = 3 per line; n = 6 per group) + SEM. Student’s t-test was applied for statistical analysis (^*^p < 0.05, ^***^p < 0.001). **(m)** Effect of oxidative stress on FTD and Ctrl astrocyte viability as analyzed by measurement of lactate dehydrogenase (LDH) release after 48 h of rotenone treatment. Data are represented as mean of eight replicates per group (n = 4 per line) + SEM. Student’s t-test was applied for statistical analysis (^*^p < 0.05).

Next, we differentiated FTD and Ctrl NPCs into mature astrocytes using a newly developed protocol spanning 90 days of differentiation and involving overexpression of SOX10 as well as application of fetal calf serum (FCS) and several neural growth and neurotrophic factors such as insulin-like growth factor (IGF), platelet-derived growth factor (PDGF) and ciliary neurotrophic factor (CNTF) during different windows of astrocytic differentiation (Fig. 2a–e; Fig. S2a,b). Both FTD and Ctrl astrocytes showed a multipolar cell morphology, expressed glial fibrillary acidic protein (GFAP; Fig. 2a), and appeared at high purity and at comparable percentages in differentiated cultures (79.3 ± 1,4% up to 90.0 ± 4.0%; Fig. 2c). In contrast to NPC-derived neurons, FTD and Ctrl astrocytes were capable of taking up L-glutamate from the culture medium, which occurred to similar extents in all FTD and Ctrl lines (Fig. 2d; Fig. S2a). FTD and Ctrl astrocytes also carried a comparable potential to propagate calcium waves upon mechanical stimulation demonstrating successful generation of functional astrocytes in both groups (Fig. S2b). We also examined the expression of astrocyte marker genes such as *GFAP*, *ALDH1L1*, *SLC1A2*, *SLC1A3* and *S100β* in FTD and Ctrl astrocytes via qRT-PCR (Fig. 2e; Fig. S6a). This analysis revealed similar expression profiles, except for *S100β*, which was more highly expressed in FTD astrocytes. Altogether, these findings indicated that overall astrocytic differentiation was not impaired in differentiated FTD NPCs.

### FTD astrocytes show disease-associated changes in *TAU* expression, increased vulnerability to oxidative stress and increased protein ubiquitination

Since patients with the N279K mutation in *MAPT* show TAU pathology not only in neurons but also in astrocytes^4^, we next examined TAU expression in our FTD and Ctrl astrocyte cultures (Fig. 2f,g; Fig. S2c,d,f,g). While we did not observe any AT8^+^ p-TAU expression in either group, we detected equal levels of full-length *TAU* mRNA and equal amounts of TAU protein when comparing the FTD and Ctrl astrocyte groups (Fig. 2f; Fig. S2d,f). Significant variability in full-length *TAU* mRNA expression was observed between the Ctrl-1 and the Ctrl-2 lines, which, however, did not result in notable differences in the expression of TAU on protein level (Fig. S2c,d,f). Also, we did not detect any differences in the amount of TAU fragments in FTD and Ctrl astrocytes (Fig. S2d). This is an interesting finding since previous studies demonstrated increased fragmentation of TAU protein in N279K *MAPT* iPS cell-derived neurons^10^ and also in iPS cell-derived neurons carrying the A152T *MAPT* variant, another pathogenic FTD-causing mutation^11^. These observations pointed towards cell-type specific processing of TAU protein in neurons and astrocytes in mutant *MAPT*-associated FTD. Notably, our FTD neurons and FTD astrocytes expressed significantly increased levels of exon 10-containing *4R-TAU* isoforms, which are usually expressed at equal levels with *3R-TAU* isoforms (devoid of exon 10) in the normal brain but which are significantly increased in the brains of patients with N279K *MAPT* (Fig. 2g; Fig. S6b)^4^. This observation thus validated our stem cell model of FTD and showed at the same time that N279K *MAPT* neurons and N279K *MAPT* astrocytes shared similar pathological splicing of *MAPT*. Further characterization of *4R-TAU* in our FTD astrocytes revealed that it is mainly comprised of the *4R/0N-TAU* isoform lacking exons 2 and 3, while, in contrast to the adult human brain, *4R/1N-TAU* (containing exon 2) and *4R/2N-TAU* (containing exons 2 and 3) were not expressed (Fig. S2g). These findings may point towards a more immature splicing of TAU in NPC-derived astrocytes and are in line with observations that the fetal form of TAU, which lacks exons 2 and 3, is also the earliest isoform that is expressed in human pluripotent stem cell-derived neural cells during differentiation *in vitro*^12^,^13^.

When examining our astrocyte cultures, we realized that the FTD astrocytes appeared much larger than Ctrl astrocytes (Fig. 2a). These changes were also clearly seen in Phalloidin staining, which were applied to visualize the cytoskeleton in astrocytes (Fig. 2b). A quantitative analysis confirmed increased cell size of FTD astrocytes and also showed that their N/C (nucleus/cytoplasm) ratio was significantly reduced compared to Ctrl astrocytes (Fig. 2h,j; Fig. S6c,e). A significant difference in nuclear size could not be detected (Fig. 2i; Fig. S6d). We also measured mildly, though significantly, elevated levels of GFAP protein within FTD astrocytes, while *GFAP* mRNA was not elevated in these cells (Fig. 2e; Fig. S2e,f). Cell hypertrophy and increased levels of GFAP are also seen in activated astrocytes in various neurodegenerative diseases, which can occur as a result of chemokine stimulation or exposure to molecular triggers such as ATP or β-amyloid^7^. Since such extrinsic stimuli were absent in our model of FTD, we concluded that N279K *MAPT* astrocytes carried particular intrinsic programs to pathological remodeling.

To characterize these programs further, we analyzed stress-associated pathways in FTD astrocytes (Fig. 2k–m; Fig. S2h–k). We first examined the expression of p-PERK and BiP in mature astrocytes, both of which are closely linked to the unfolded protein response (UPR), a stress response due to an accumulation of misfolded proteins within the endoplasmic reticulum (ER)^14^. While we have previously demonstrated UPR activation in iPS cell-derived neurons carrying N279K *MAPT*^10^, we did not observe an increase in p-PERK and BiP in N279K *MAPT* FTD astrocytes indicating neural cell type-specific differences in UPR in FTD (Fig. S2h,i). Next, we performed western blot analysis to determine the amount of ubiquitinated proteins in FTD and Ctrl astrocytes as a means of proteasome-associated protein degradation, which plays a significant role in neural degeneration in a variety of neurodegenerative diseases including FTD (Fig. 2k,l; Fig. S5a,b; Fig. S6f)^15^. This analysis revealed significantly increased levels of ubiquitinated proteins in N279K *MAPT* astrocytes and thus an alteration of protein degradation in these cells (Fig. 2k,l; Fig. S6f). Further analysis of stress susceptibilities in FTD astrocytes revealed an increased vulnerability to oxidative stress, despite differences in the vulnerability of individual FTD clones (Fig. 2m; Fig. S6g). Application of 1 μM rotenone, a complex I inhibitor of the mitochondrial respiratory chain, resulted in increased cell death with significantly increased release of lactate dehydrogenase (LDH) in FTD compared to Ctrl astrocytes (Fig. 2m; Fig. S6g). Quantification of remaining, surviving astrocytes, which expressed the apoptotic marker cleaved CASPASE-3, showed a tendency, though not a significant increase in cleaved CASPASE-3 positivity in FTD astrocytes 48 hours after application of rotenone (Fig. S2j,k).

Altogether, these findings showed altered protein degradation and an increased susceptibility to respiratory stress but no activated UPR in N279K *MAPT* astrocytes.

### FTD astrocytes show alterations in whole genome expression profiles

**Figure 3:**
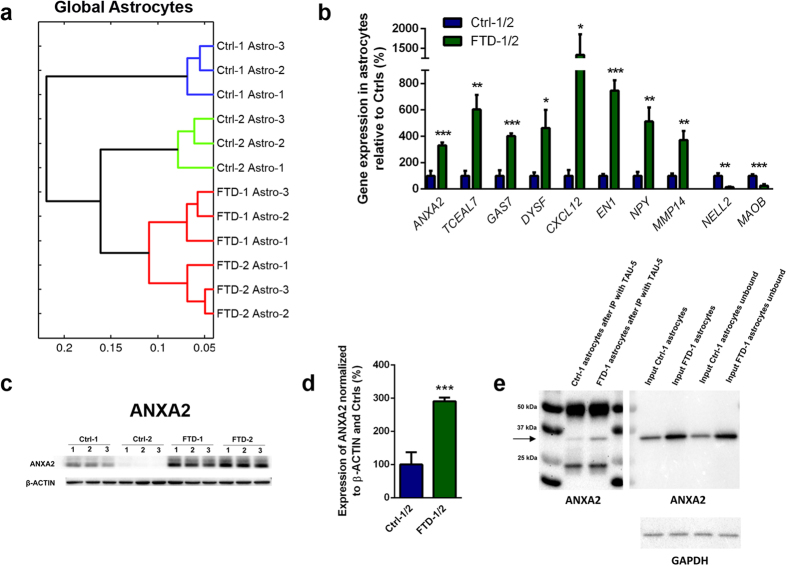
Transcriptome profiles in FTD and control astrocytes and identification of ANXA2 as an upregulated protein in FTD astrocytes interacting with TAU. **(a)** Hierarchical cluster analysis of whole genome expression profiles in FTD and Ctrl astrocytes. **(b)** qRT-PCR expression analysis of differentially expressed genes in FTD and Ctrl astrocytes. **(c)** Western blot expression analysis of ANXA2 protein in FTD and Ctrl astrocytes. β-ACTIN was used as loading control. Independent replicates are shown for each line. Images have been cropped using Photoshop software. Full-length blots are presented in Supplementary Fig. S5c,d. **(d)** Quantification of ANXA2 protein expression in astrocytes. Data in panels b and d are represented as mean of replicates from three independent differentiation experiments (n = 3 per line; n = 6 per group) + SEM. Student’s t-test was performed for statistical analysis (^*^p < 0.05, ^**^p < 0.01, ^***^p < 0.001). **(e)** Co-immunoprecipitation to evaluate interaction of ANXA2 and TAU. TAU-5 antibody was used to immunoprecipitate TAU from protein extracts of Ctrl-1 and FTD-1 astrocytes. TAU-5 immunoprecipitates were analyzed by western blot and probed by ANXA2 antibody. Input fractions were included as controls. GAPDH was used as loading control. Arrow indicates ANXA2 protein. Images have been cropped using Photoshop software. Full-length blots are presented in Supplementary Fig. S5e–g.

Next, we performed microarray analysis on FTD and Ctrl astrocytes to determine if global gene expression was altered in N279K *MAPT* astrocytes (Fig. 3a,b; Fig. S3a). We found that whole genome expression profiles differed significantly between FTD and Ctrl astrocytes, as they separated clearly in the heat map (Fig. S3a) and formed separate clusters in the hierarchical cluster analysis (Fig. 3a). We also observed that the distance between FTD astrocytes and isogenic Ctrl astrocytes (Ctrl-2) was much shorter than the distance between FTD astrocytes and genetically unrelated, non-isogenic Ctrl astrocytes (Ctrl-1; Fig. 3a) demonstrating that global gene expression in Ctrl cells was largely dependent on their genetic background (Fig. 3a, Fig. S3a). We next investigated the genes, which were differentially up- or downregulated in FTD astrocytes. Functional annotation of downregulated genes were related to Gene Ontology (GO) terms such as integral to plasma membrane (*GPRC5C*, *HLA-DRB5*) and multicellular organism development (*DLK1*, *SEMA5A*), while upregulated genes were associated with neurological system processes (*CHRNA1*, *CRYZ*, *EYA1*, *NPY*, *PCDHB5*), synapse organization and biogenesis (*CHRNA1*, *PCDHB5*) and synaptic transmission (*CHRNA1*, *NPY*, *PCDHB5*). These were significant observations since astrocytes play an important role in the formation and strengthening of synaptic connections in the healthy human brain^7^. Additional downregulated genes included *NELL2* and *MOAB*, which have been associated with cellular stress responses (Fig. 3b; Fig. S6h). Additional upregulated genes included genes that have been linked to astrocyte migration (*MMP14*), neural survival (*EN1, NPY*), microtubule maintenance and TAU-associated pathology in AD (*GAS7*), protein accumulation in AD (*DYSF*) and regulation of NF-kappaB, which is involved in various cellular processes including activation of cultured astrocytes (*TCEAL7*, *CXCL12;* Fig. 3b; Fig. S6h)^16^,^17^,^18^,^19^. Notably, we also found a significant upregulation of Annexin A2 (*ANXA2*) in FTD astrocytes, which we confirmed at the protein level (Fig. 3b–d; Fig. S6h,i). ANXA2 is a calcium-dependent multifunctional phospholipid-binding protein associated to the plasma membrane and endosomal compartments^20^. It is upregulated in hypoxia^20^ and in reactive astrocytes^21^ and was identified as an interaction partner of TAU protein, linking membranous components to the cytoskeleton^22^. In the latter study, it was also demonstrated that TAU carrying the R406W *MAPT* mutation, another *MAPT* mutation causing FTD, led to impaired neurite outgrowth when overexpressed in PC12 cells and that wildtype TAU, but not TAU carrying the R406W mutation, interacts with ANXA2 protein in a heterologous yeast expression system^22^. Thus, we performed immunoprecipitation to evaluate binding of N279K TAU and non-mutant TAU to ANXA2 in our FTD and Ctrl astrocytes, respectively (Fig. 3e). This analysis revealed that a small fraction of TAU binds to ANXA2 in human NPC-derived astrocytes and that N279K mutant *MAPT* did not cause an impaired binding of TAU to ANXA2 in FTD astrocytes (Fig. 3e). This is an important finding since FTD astrocytes expressed significantly increased amounts of ANXA2 protein with TAU-binding ability, potentially causing a functional disequilibrium in FTD astrocytes.

To next determine if expression patterns of dysregulated genes were specific to FTD astrocytes, we tested expression of the aforementioned genes in FTD NPC-derived neurons (Fig. S3b). While expression profiles of most of these genes did not differ between FTD and Ctrl neurons, we observed an upregulation of *ANXA2* and of *TCEAL7* and a downregulation of *NELL2* in FTD neurons similar to FTD astrocytes indicating that these genes may play an important role in both neural cell types in FTD.

Next, we determined expression patterns of aging-associated genes in FTD and Ctrl astrocytes since aging is a strong risk factor for neural degeneration (Fig. S3c,e). First, we analyzed expression levels of the aging-related key genes *RANBP17*, *SIRT1*, *SIRT6*, *TERF1*, *TERF2* and *CDKN1C*, which did not differ between FTD and Ctrl astrocytes but which were in part decreased in FTD neurons compared to Ctrl neurons (*TERF1* and *CDKN1C*; Fig. S3c,d). Thus, we extended our analysis and tested for the expression of 255 genes in FTD and Ctrl astrocytes that are significantly upregulated in the forebrain of aged individuals^23^. Comparative hierarchical cluster analysis of the expression of these genes revealed robust separation of FTD astrocytes from Ctrl astrocytes indicating an intrinsic difference in aging patterns in these two groups (Fig. S3e). In addition, FTD and Ctrl astrocytes separated as a group from FTD and Ctrl NPCs, consistent with a different level of maturation in differentiated astrocytes compared to NPCs (Fig. S3e).

### Co-culture of healthy Ctrl neurons with FTD or Ctrl astrocytes reveals alterations in stress responses and changes in gene expression in FACS-isolated neurons

**Figure 4:**
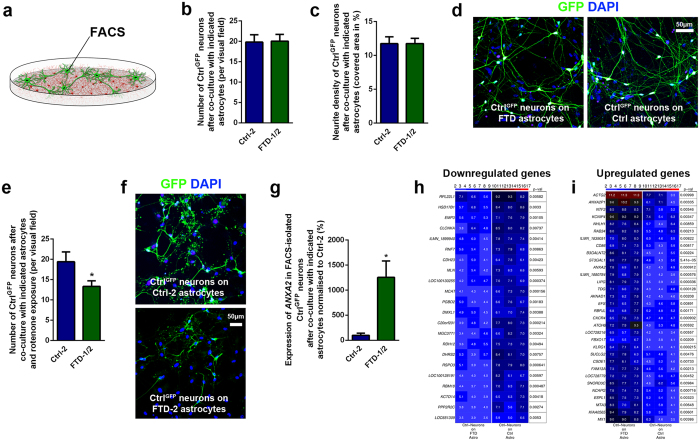
Co-culture of control neurons with FTD astrocytes induces alterations in stress response and gene expression profiles. **(a)** Schematic drawing demonstrating the co-culture of healthy Ctrl^GFP^ neurons (green) with either FTD or Ctrl astrocytes (red). **(b,c)** Quantification of the number of Ctrl^GFP^ neurons (**b**) and their neurite density (**c**) after co-culture with either FTD or Ctrl astrocytes. Data are represented as mean of three replicates per line from three independent co-culture experiments (n_Ctrl_ = 3, n_FTD_ = 6) + SEM. **(d)** Fluorescence images showing Ctrl^GFP^ neurons cultured on either FTD or Ctrl astrocytes. Nuclei were counterstained with DAPI (blue). Scale bar = 50 μm. **(e)** Effect of oxidative stress on Ctrl^GFP^ neurons after co-culture with either FTD or Ctrl astrocytes as analyzed by the number of surviving neurons after 48 h of rotenone treatment. Data are represented as mean of three replicates per line from three independent co-culture experiments (n_Ctrl_ = 3, n_FTD_ = 6) + SEM. Student’s t-test was performed for statistical analysis (*p < 0.05). **(f)** Fluorescence images showing Ctrl^GFP^ neurons cultured on either FTD or Ctrl astrocytes 48 h after treatment with rotenone. Nuclei were counterstained with DAPI (blue). Scale bar = 50 μm. **(g–i)** Ctrl^GFP^ neurons were isolated from FTD or Ctrl astrocytes via FACS and expression analyses were performed. **(g)** qRT-PCR expression analysis of *ANXA2* in FACS-isolated Ctrl^GFP^ neurons after co-culture with either FTD or Ctrl astrocytes. Data are represented as mean of three replicates per line from three independent co-culture experiments (n_Ctrl_ = 3, n_FTD_ = 6) + SEM. Student’s t-test was performed for statistical analysis (*p < 0.05). **(h,i)** Heat maps of differentially downregulated (**h**) and upregulated (**i**) genes in isolated Ctrl^GFP^ neurons with p-values.

We next tested if healthy neurons could change their neural programs when co-cultured with N279K *MAPT* astrocytes (Fig. 4; Fig. S4). To this end, we used a lentiviral expression system to label Ctrl neurons with green fluorescent protein (GFP; Ctrl^GFP^ neurons differentiated from Ctrl-1 NPCs). Ctrl^GFP^ neurons were co-cultured with either FTD or Ctrl astrocytes for 3 weeks and cell survival, neurite outgrowth, synaptic coverage, respiratory stress responses and transcriptome profiles were determined in GFP^+^ neurons. While overall survival, neurite density and synaptic coverage of Ctrl^GFP^ neuronal profiles were not altered by co-culture with either astrocyte type (Fig. 4b–d; Fig. S4a,b and S6j,k), we observed an increased susceptibility of Ctrl^GFP^ neurons to rotenone-induced oxidative stress after co-culture with N279K *MAPT* astrocytes (Fig. 4e,f; Fig. S6l). 48 hours after application of rotenone, we found a significantly lower number of Ctrl^GFP^ neurons when co-cultured on FTD astrocytes compared to Ctrl^GFP^ neurons co-cultured on Ctrl astrocytes, indicating an increased vulnerability of neurons in the presence of FTD astrocytes (Fig. 4e,f; Fig. S6l). Also, a small number of the remaining surviving neurons expressed cleaved CASPASE-3 with a tendency, though not a significant change towards higher numbers in neurons co-cultured with FTD astrocytes (Fig. S4c,d). An analysis of the neuronal subtypes before and after rotenone treatment showed that the relative percentage of GABAergic and dopaminergic neurons, the most abundant neuronal subtypes obtained with our differentiation protocol^10^, was not changed in Ctrl^GFP^ neurons when cultured on FTD astrocytes (GABAergic neurons: 64.0 ± 11.3% before and 61.5 ± 23.3% in surviving neurons after rotenone; dopaminergic neurons: 34.7 ± 31.1% before and 22.7 ± 26.2% in surviving neurons after rotenone). In addition, the regional identity of Ctrl^GFP^ neurons along the rostral-caudal axis was not altered by the presence of either FTD or Ctrl astrocytes and corresponds to brain stem domains, which are significantly affected in FTD caused by mutant N279K *MAPT* (Fig. S4e)^24^. Also, co-cultured neurons and astrocytes carried comparable regional profiles, consistent with the fact that both neural cell types had been derived from the same NPC population with similar regional programs (Fig. S4e)^9^. Altogether, these findings indicated that FTD astrocytes could render neighboring neurons more vulnerable to respiratory stress. Interestingly, these changes seemed to require direct cell-cell contact since conditioned medium from FTD astrocytes did not increase vulnerability of rotenone-challenged Ctrl^GFP^ neurons (Fig. S4f–h).

We extended our analysis to examining gene expression profiles in Ctrl^GFP^ neurons by isolating GFP^+^ neuronal profiles from FTD or Ctrl astrocytes via fluorescence-activated cell sorting (FACS; Fig. 4a,g–i). First, we determined expression levels of those genes in neurons, which were dysregulated in FTD astrocytes (Fig. 3b) and partially also in FTD neurons (Fig. S3b). This analysis revealed only one gene, *ANXA2*, to be significantly upregulated in neurons after co-culture with FTD astrocytes while expression levels of the other previously identified genes (*TCEAL7*, *GAS7*, *DYSF*, *CXCL12*, *NPY*, *MMP14*, *NELL2*, *MAOB*) were comparable (Fig. 4g; Figs S4i and S6m). These observations indicated that ANXA2 may also play an important role in astrocyte-neuron-crosstalk in the context of FTD. Next, whole transcriptome profiles were determined in isolated Ctrl^GFP^ neurons, which showed that global gene expression differed only mildly between FTD and Ctrl astrocyte-co-cultured neurons with no clear separation of cells in the global cluster analysis (Fig. S4j). However, when determining differentially regulated genes in these two neuronal populations, we found significant changes between both groups (Fig. 4h,i; Fig. S4k–m). Functional annotation of downregulated genes in Ctrl^GFP^ neurons co-cultured with FTD astrocytes were related to negative regulation of cellular process (*DHRS2*, *EMP3*, *RNF7*), hormone metabolic process (*DHRS2*, *RDH12*), system process (*CDH23*, *CLCNKA*, *RDH12*) and cellular lipid metabolic process (*DHRS2*, *MCAT*, *RDH12*) (Fig. 4h; Fig. S4k). In line with our previous observations, upregulated genes in Ctrl^GFP^ neurons co-cultured with N279K *MAPT* astrocytes were associated with apoptosis (*CXCR4*, *ESPL1*, *MX1*), response to stress (*CXCR4*, *KLRG1*, *TDG*), system development (*ANXA2*, *CSDE1*, *NHLH1*, *NTF3*) and signal transduction (*CXCR4*, *EFS*, *KLRG1*, *MX1*, *NTF3*, *RBPJL*) (Fig. 4i; Fig. S4l,m). These findings indicated that FTD astrocytes have the ability to significantly influence neural programs in previously healthy neurons and demonstrate astrocyte-mediated non-cell-autonomous changes in FTD, which have similarly been described as an important pathogenic component in other neurodegenerative diseases such as ALS^5^,^6^.

The CRISPR/Cas9 system was originally discovered as a bacterial immune response to foreign DNA and was subsequently remodeled to allow double strand breaks in specific regions of mammalian genomes^25^,^26^,^27^. We demonstrate that CRISPR/Cas9 genome editing can successfully be applied in NPCs to analyze disease phenotypes in FTD caused by mutant *MAPT*. Homologous recombination in human pluripotent stem cells (PSCs) has been feasible, albeit with low frequencies of homology directed repair (7 HDR/350 human ES clones, 0.02%)^28^. While most mammalian applications of CRISPR/Cas9 genome editing methodology have been described in PSCs, one report recently extended the application of CRISPR/Cas9 to directly ablate genes in human CD34^+^ blood stem/progenitor cells and CD4^+^ T cells^29^. This study reported CRISPR/Cas9-directed knockout frequencies in somatic cells ranging from 22% to 40% in human erythroleukemic K562 cells and 27% in human CD34^+^ stem/progenitor cells^29^. Here, we demonstrate efficient homologous recombination in NPCs after CRISPR/Cas9 vector application at a frequency of 12%. Genome editing in NPCs may provide certain advantages over genetic correction in PSCs, as NPCs comprise robust, easily expandable somatic progenitor populations, which are already committed to neural differentiation^8^,^9^,^10^,^30^. We foresee that our NPC-targeted CRISPR/Cas9 genome editing approach can successfully be applied to model neurodegenerative diseases other than FTD *in vitro*.

Our study demonstrates profound intrinsic pathologic changes in N279K *MAPT* astrocytes in regard to cell morphology, TAU expression, protein ubiquitination, susceptibility to oxidative stress and whole genome expression profiles. N279K *MAPT* astrocytes also modified neural programs and increased stress responses in co-cultured, previously healthy neurons. This increased vulnerability was associated with an upregulation of stress- and apoptosis-related genes including the cytokine receptor *CXCR4*, which is widely expressed in neurons and glial cells in the central nervous system and which, when downregulated, leads to significant extension in lifespan and improved motor function in SOD1(G93A) ALS mice^31^. Upregulated genes in neurons co-cultured on FTD astrocytes also included *ANXA2*, a TAU-binding protein, which was also upregulated in our FTD astrocytes and also FTD neurons. Interestingly, an upregulation of ANXA2 has also been described in the frontal lobe of patients with FTD with ubiquitin-positive inclusions^32^, further emphasizing a potentially essential role of ANXA2 in the pathogenesis of FTD. Additional studies such as an siRNA or CRISPR/Cas9-mediated knockdown of *ANXA2* in N279K *MAPT* FTD neurons and astrocytes may be very useful to further evaluate mechanisms by which ANXA2 influences cellular integrity in neural cells in the context of mutant *MAPT*-associated FTD. While our study examines astrocytes of independent NPC clones from one patient carrying the N279K *MAPT* mutation, it would be very interesting to expand the study to astrocytes from additional FTD patients including patients that harbor different pathogenic mutations in *MAPT*.

Our human neural stem cell model of FTD does not only provide a suitable platform to identify disease phenotypes in patient-derived astrocytes, but it also serves as an invaluable tool for future, therapy-oriented drug screens on FTD astrocytes, which may significantly influence neuronal degeneration in FTD.

## Methods

### Genome editing of human NPCs

The Cas9 and single guide RNA (sgRNA) harboring vector pX260 (Addgene plasmid #42229) was used to edit the N279K *MAPT* mutation on chromosome 17 (17q21.1) in FTD iPS cell-derived NPCs. sgRNAs were designed to target exon 10 of *MAPT* using the Target Finder Software (Feng Zhang Laboratory, MIT, Cambridge, USA, http://crispr.mit.edu.; MAPT10_1: 5′-GTACTCACACTGCCGCCTCC-3′; MAPT10_2: 5′-AGGCGTCCTTGCGAGCAAGC-3′). Annealed oligos were ligated into pX260. A 156-nt single-stranded DNA oligonucleotide (ssODN) containing the wildtype nucleotide sequence (TTGCGAGCAA GCAGGCGGGT CCAGGGTGGC GTGTCACTCA TCCTTTTTTC TGGCTACCAA AGGTGCAGAT AATTAATAAG AAGCTGGATC TTAGCAACGT CCAGTCCAAG TGTGGCTCAA AGGATAATAT CAAACACGTC CCGGGAGGCG GCAGTG) was used as template for homology directed repair (HDR). The vector (1.5 μg of pX260- MAPT10_1 and 1.5 μg or 2 μg of pX260- MAPT10_2) and the ssODN (1.5 μg or 2 μg, respectively) were co-transfected into NPCs using Fugene 6 (Promega) as transfection reagent according to the manufacturer´s manual. After selection for transfected cells with addition of 5 μg/ml puromycin, emerging single cell colonies were transferred to 96-well plates for further expansion. Genomic DNA was prepared by isopropanol precipitation and successful HDR-mediated mutation correction was assessed by amplification of an exon 10 sequence of *MAPT* using the PCR primers (5′-CGAGCAAGCAGCGGGTCC-3′) and (5′-GTACGACTCACACCACTTCC-3′) and consecutive restriction digests with *Mbo*II, distinguishing wildtype from mutant *MAPT*, followed by genomic sequencing. To ensure that the gene-corrected cell lines originated from N279K *MAPT* cells, short tandem repeat (STR) loci were compared using the PowerPlex Fusion System (Promega).

### Astroglial differentiation of NPCs

For astroglial differentiation, NPCs were transduced with different dilutions of lentivirus containing the pLEX307 vector, which was a gift from David Root (Addgene plasmid # 41392), expressing SOX10. Virus was produced by co-transfection of 293T cells with pLEX307-SOX10 and packaging plasmids, and viral particles were subsequently concentrated by ultracentrifugation and stored at −80 °C. Cells were infected with lentivirus for 24 h and in the presence of 5 μg/ml protamine sulfate (Sigma). Two days after lentiviral transduction, medium was changed to DMEM-F12 with 1:100 N2 supplement (Invitrogen), 1:100 B27 supplement lacking vitamin A (Invitrogen), 1% penicillin/streptomycin/glutamine, 1 μM SAG, 10 ng/ml PDGF (Peprotech), 2.5 ng/ml bFGF (Peprotech), 10 ng/ml NT3 (Peprotech), 10 μg/ml IGF (Sigma), 200 μM ascorbic acid (AA, Sigma), 1:1000 Trace Element B (Corning), 0.5 μM LDN 193189 (Axon Medchem) and additionally with 5 μg/ml puromycin for one week to remove non-transduced cells. Cells were split once a week at ratios of 1:2 to 1:5 after detachment with accutase. After 45 days, medium was changed once more to astrocyte differentiation medium consisting of DMEM-F12 with 1:100 N2 supplement, 1:100 B27 supplement lacking vitamin A, 1% penicillin/streptomycin/glutamine, 4% FCS (Biochrom), 10 μg/ml IGF, 10 ng/ml CNTF (Peprotech), 200 μM AA and 50 μM dbcAMP (Sigma). Astrocyte cultures were split once a week using accutase and replated on fresh matrigel-coated plates. After 40 to 45 days in astrocyte differentiation medium cells were either fixed in 4% paraformaldehyde (PFA) or were lysed in RLT buffer (Qiagen) or in ice-cold RIPA buffer.

### Glutamate uptake assay

The glutamate clearance capacity of astrocyte cultures was assessed using the Glutamate Colorimetric Assay Kit (BioVision, USA) according to the manufacturer’s instructions and in comparison to neuronal cultures. Briefly, cells were plated at a density of 10^5^ cells in matrigel-coated 12-well plates one day prior to performing the assay. 24 h after plating, cells were washed once with HBSS (Gibco) and subsequently incubated with HBSS containing 500 μM glutamate. After incubation for 2 h at 37 °C, 20 μl of cell supernatant or medium control were transferred into a 96-well plate, diluted with assay buffer to a volume of 50 μl and incubated with 100 μl of the reaction mix for 30 min at 37 °C. Absorbance of the product was measured at 450 nm using a microplate reader. For quantification of glutamate concentrations, a standard curve was created in each assay based on measurement of cell-free HBSS containing known glutamate concentrations.

### Visualization of calcium signaling

For visualization of intercellular calcium signal transmission, astrocytes were plated on matrigel-coated glass coverslips at a density of 3 × 10^4^ cells per well of a 12-well plate. Three days after plating, astrocytes were loaded with the fluorescent calcium indicator Oregon Green 488 BAPTA-1 (Thermo Fisher Scientific) for at least 45 min at 37 °C. Glass coverslips were subsequently placed in a recording chamber mounted to an inverted microscope (Observer.A1, Zeiss) and superfused at 2 ml/min at RT with a bath solution containing (in mM:) NaCl 130, NaH_2_CO_3_ 10, KCl 3, MgCl_2_ 1.5, CaCl_2_ 1.5, glucose 11, HEPES 10; pH 7.3 adjusted with NaOH. To evoke intercellular transmission of calcium signals, single cells were mechanically stimulated with a glass pipette controlled by a micromanipulator (MPC-200, Sutter Instrument). Calcium waves were visualized and recorded with a camera (Orca Flash4.0), a LED-light source and VisiView software (Visitron Systems). Data were analyzed with ImageJ (National Insitute of Health, USA).

Please see the Supplementary Information for further details. Student’s t-test was performed for statistical analysis unless otherwise specified.

## Additional Information

**Accession codes:** The data discussed in this publication have been deposited in NCBI’s Gene Expression Omnibus and are accessible through GEO Series accession number GSE795. (http://www.ncbi.nlm.nih.gov/geo/query/acc.cgi?token=exsruggmzhylxcv&acc=GSE79557).

**How to cite this article:** Hallmann, A.-L. *et al*. Astrocyte pathology in a human neural stem cell model of frontotemporal dementia caused by mutant TAU protein. *Sci. Rep.* **7**, 42991; doi: 10.1038/srep42991 (2017).

**Publisher's note:** Springer Nature remains neutral with regard to jurisdictional claims in published maps and institutional affiliations.

## Supplementary Material

Supplementary Information
