# Supplementary material for: Astrocyte pathology in a human neural stem cell model of frontotemporal dementia caused by mutant TAU protein
Source: Sci Rep. 2017 Mar 3;7:42991. doi: 10.1038/srep42991 (PMC5335603; doi:10.1038/srep42991)
Supplement: Supplementary Information [file srep42991-s1.docx]

**Supplementary Information**

**Astrocyte pathology in a human neural stem cell model of frontotemporal dementia caused by mutant TAU protein**

Anna-Lena Hallmann, Marcos J. Araúzo-Bravo, Lampros Mavrommatis, Marc Ehrlich, Albrecht Rӧpke, Johannes Brockhaus, Markus Missler, Jared Sterneckert, Hans R. Schöler, Tanja Kuhlmann, Holm Zaehres, Gunnar Hargus

**Supplementary Methods**

**Derivation and culturing of human neural progenitor cells**

Fibroblasts from an individual carrying the N279K mutation in *MAPT*, here referred to as FTD patient, were obtained from the Coriell Institute for Medical Research, which had received informed consent from the donor. Fibroblasts had been reprogrammed into iPS cells using the lentiviral SF-OSKM-tomato expression vector as previously described^1^. All procedures were approved and were carried out in accordance with approved guidelines (MPI 53.02.01-M-1.26/10; Bezirksregierung Düsseldorf). Neural progenitor cells (NPCs) were differentiated from FTD iPS cells using a well-established differentiation protocol^1-3^. NPCs were cultured in expansion medium consisting in equal parts of DMEM-F12 (Invitrogen) and neurobasal medium (Invitrogen) with 1:200 N2 supplement (Invitrogen), 1:100 B27 supplement lacking vitamin A (Invitrogen), 1% penicillin/streptomycin/glutamine, 3 µM CHIR99021 (Axon Medchem), 0.5 µM SAG (Cayman Chemical) and 150 µM ascorbic acid (AA; Sigma). Cells were grown on matrigel-coated (Matrigel, growth factor reduced, high concentration; BD Bioscience) 12-well plates (Nunc) and split once a week at ratios of 1:15 to 1:20 by treatment with accutase (Sigma).

**Karyotype analysis of FTD NPCs and gene-corrected control NPCs**

Karyotype analysis of isogenic control NPCs was performed as previously described^1^. Briefly, confluent cultures of NPCs were incubated with 0.3 mg/ml colcimid (KaryoMAC; Invitrogen) for 3 h, washed with PBS and digested to single cells by treatment with trypsin-EDTA in DMEM. Singularized cells were centrifuged and subsequently resuspended in KCl solution (75 mM). Following incubation for 7 min at room temperature, cells were spun down and resuspended in ice-cold fixation solution (3:1 methanol/acetic acid) and left shaking. Fixed cells were centrifuged once more and taken up in fresh fixative and incubated for another 20 min at 4°C. Different dilutions of cells were dropped onto glass slides (Menzel Gläser, Thermo Scientific) for analysis. Chromosomes were GTG-banded by standard procedures and metaphase spreads were analyzed using a Zeiss AxioScop and the Cytovision software (Applied Imaging Corporation).

**Immunocytochemistry**

For immunocytochemical staining, cells were fixed in 4% PFA in PBS for 20 min at RT. Fixed cells were permeabilized by three washing steps with 0.2% Triton X-100 (Sigma) in PBS before blocking, which was performed by incubating the cells with 10% normal goat serum (NGS) in PBS for 1 h at RT. Primary Antibodies (GFAP: Dako, Z0334, rabbit, 1:4000; Synaptophysin: Dako, M0776, mouse, 1:500; GFP: Abcam, ab6556, rabbit, 1:2000; MAP2: Santa Cruz, SC-20172, rabbit, 1:1000; βIII-Tubulin: Covance, MMS-435P, mouse, 1:750; AT8: Thermo Scientific, MN1020, mouse, 1:150; Nestin: R&D Systems, MAB1259, mouse, 1:300; SOX1: R&D Systems, AF3369, goat, 1: 150; cleaved CASPASE-3 (Asp175): Cell Signaling, #9661, rabbit, 1:200; TH: Pel-Freez, P40101-150, rabbit, 1:500, GABA-RB: Sigma, SAB4501062, rabbit, 1:100) were applied overnight at 4 °C in blocking solution. Following three washing steps with PBS, cells were incubated with Alexa Fluor conjugated secondary antibodies diluted in blocking solution for 1 h at RT. Secondary antibody incubation was followed by three washing steps with PBS with the second washing step containing a DAPI staining for nuclei. Cells on glass coverslips were mounted in Shandon Immu-Mount medium (Fisher Scientific) and visualized on a Zeiss LSM700 confocal microscope. For Phalloidin staining, permeabilized cells were incubated with Alexa Fluor® 488 Phalloidin (Thermo Fisher Scientific, A12379) for 30 min at RT. Follwing three washing steps, cells were mounted in Roti®-Mount FluorCare DAPI (Roth).

**Quantitative Real-time PCR**

The RNeasy mini kit (Qiagen) was used for the isolation of total RNA from cell lysates. RNA extraction was performed according to the manufacturer´s protocol, including an on-column DNA digestion (RNase free DNase Set; Qiagen). cDNA was generated by reverse transcription of isolated RNAs using the High Capacity cDNA reverse Transcription Kit (Applied Biosystems). 0.375- 4 ng cDNA were subsequently used for qRT-PCR reactions performed on a StepOne Plus real time cycler (Applied Biosystems) with the Power SYBR Green PCR master mix (Applied Biosystems). Specificity of the primers used for qRT-PCR reactions was determined beforehand, by agarose gel electrophoresis. qRT-PCRs were run as follows: 2 min at 50°C, 10 min at 95°C, 40 cycles of 15 sec at 95°C and 1 min at 55-60°C. Expression levels were calculated applying the 2^-∆∆ct^ method and normalizing to *GAPDH* and the biological reference sample. Sequences of primers used in this study are listed in Table S1.

**Whole genome expression analysis and microarray data processing**

For whole genome expression analyses, RNA was isolated from cell lysates using the RNeasy kit with on-column DNA digestion. 300 ng of total RNA was used as input for cRNA synthesis with the linear amplification protocol (Ambion) comprising the synthesis of T7-linked double stranded cDNA and *in vitro* transcription for 12 h incorporating biotin-labeled nucleotides. Following purification, labeled cRNA samples were hybridized onto HumanHT-12 v4 expression Beadchips (Illumina) for 18 h following the manufacturer´s protocol. The beadchips were washed as recommended and subsequently stained with streptavidin-Cy3 (GE Healthcare) and scanned using the iScan reader (Illumina) and the associated software. All samples were hybridized as biological replicates.

Data were processed by mapping bead intensities to the corresponding gene information using BeadStudio 3.2 (Illumina) and background correction was achieved by applying the Affymetrix Robust Multiarray Analysis (RMA) background correction model^4^. Variance stabilization was performed using log2 scaling. Gene expression normalization was calculated using the quantile method implemented in the lumi package of R-Bioconductor. In-house developed functions in MATLAB were utilized for data post-processing and the generation of graphical representations. Hierarchical clustering analysis of genes and samples was performed with the one minus the sample correlation metric and the Unweighted Pair-Group Method using Average (UPGMA) linkage method as previously described^5^.

**Western blot**

For protein analyses, cells were lysed on ice with RIPA-buffer containing protease inhibitors (Roche) and cell lysates were incubated for 5 min at 95°C. Protein concentrations were determined using the Pierce BCA Protein Assay Kit (ThermoFisher) and equal amounts of protein per sample were mixed with 6x Laemmli buffer, incubated for 5 min at 95°C and applied on 4-12% polyacrylamide (NuPAGE, Invitrogen) gels. Following electrophoretical separation, proteins were transferred onto a PVDF membrane. Blocking was performed for 1 h at RT using either 5% milk powder or 5% BSA in TBST. Incubation with primary antibodies (GFAP: Dako, Z0334, rabbit, 1:1000; BIP: Cell Signaling, #3177, rabbit, 1:1000; p-PERK: Santa Cruz, sc-32577, rabbit, 1:1000; TAU5: Invitrogen, AHB0042, mouse, 1:1000; ANXA2: Cell Signaling, #8235, rabbit, 1:1000; Ubiquitin: Dako, Z0458, rabbit, 1: 2000; GAPDH: Sigma, G9545, rabbit, 1:200000; β-ACTIN: Sigma, A5441, mouse, 1:200000) was carried out overnight at 4°C. After washing the membrane three times with TBST, HRP- conjugated secondary antibodies were applied for 1 h at RT. After washing the membrane again three times with TBST, chemiluminescent HRP substrate solution was applied to the membrane. Proteins were detected using the ChemiDocTM XRS+ System (BioRad) and expression levels were quantified by densitometric analysis with the Image LabTM software (BioRad). For detection of TAU and p-PERK, protein samples were precipitated prior to immunoblotting. In short, volumes of protein samples corresponding to 80 µg of protein were mixed with nine times the sample volume of cold (-20°C) acetone (Roth) and incubated overnight at -20°C. The next day, samples were centrifuged (20 min/ 21000 rcf/ 4°C), the supernatant was decanted and pellets were left to dry for approximately 1 h at RT. The dried pellet was subsequently resuspended in water and 6x Laemmli buffer was added.

#### Immunoprecipitation

To investigate a potential interaction of TAU and ANXA2, total protein was extracted from FTD-1 and Ctrl-1 astrocytes using Triton X-lysis buffer (10 mM Tris-HCl, pH 7.6, 150 mM NaCl, 2 mM MgCl_2,_ 2 mM EDTA, 0.1% Triton X-100, 10% Glycerol, 1 mM PMSF, 1 tablet cOmplete, Mini, protease inhibitor (Roche), 1 tablet PhosSTOP, phophatase inhibitor (Roche), in 10 ml H_2_O). Immunoprecipitation was conducted using the Immunoprecipitation Kit - Dynabeads® Protein G (Invitrogen) according to the manufacturer´s recommendations. 50 µl Dynabeads were incubated with 5 µg TAU-5 antibody in 200 µl Ab Binding & Washing Buffer for 1 h at 4°C. Dynabeads were subsequently washed once with Ab Binding & Washing Buffer, before being incubated with 250 µg of protein extract overnight at 4°C. Unbound protein was subsequently transferred into a clean reaction tube for latter analyses and Dynabeads were washed three times with Washing Buffer. During a fourth washing step, Dynabeads were transferred into a clean reaction tube before 15 µl Elution Buffer were added. Following incubation for 20 min at 4°C, the eluate was removed from the Dynabeads and subsequently mixed with 4x loading buffer and boiled for 5 min at 95°C. Samples were immediately loaded onto gels for SDS-PAGE and later-on analyzed by western blot. All incubation steps involving Dynabeads were performed on a roller.

**Quantification of the cell and nuclear sizes of astrocytes**

The ImageJ software was used to measure the sizes of astrocytes and their nuclei. To this end, astrocytes were stained for GFAP and nuclei were counterstained with DAPI. Images of immunofluorescent staining were taken using the Zeiss LSM700 confocal microscope. Outlines of GFAP^+^ cells and of their nuclei were manually encircled and sizes of the encircled areas were determined with the ImageJ software and the scale bar incorporated in the immunofluorescence images.

**Determination of the *4R-TAU* isoforms expressed in astrocytes**

To determine which *4R-TAU* isoforms were expressed in cultured astrocytes, cDNA from FTD-1 astrocytes was amplified using a forward primer specific for exon 1 of the *MAPT* gene (5´CGAAGTGATGGAAGATCACG´3) and a reverse primer specific for exon 10 of the *MAPT* gene (5´CACACTTGGACTGGACGTTG´3). The size of the PCR product corresponding to the 4R/0N isoform, which lacks both, exon 2 and exon 3, was calculated to be 676 bp, the size of the PCR product corresponding to the 4R/1N isoform, which contains exon 2 but lacks exon 3, was calculated to be 764 bp and the size of the PCR product corresponding to the 4R/2N isoform, which contains both, exon 2 and exon 3, was calculated to be 851 bp.

**Rotenone stress assay**

To examine the effect of rotenone (Sigma) on the viability of FTD and Ctrl astrocytes, astrocytes were plated at a density of 8 x 10^3^ cells per well into 96-well plates. After 12 days in maturation medium, 0.5 μM and 1 μM rotenone in N2 medium consisting of DMEM-F12 with 1:100 N2 supplement and 1% penicillin/streptomycin/glutamine were added to the cells for 48 h. Viability was assessed by measuring LDH leakage in the cell culture supernatant using the Cytotoxicity Detection Kit Plus (Roche). Absorbance was recorded at 490 nm and was expressed as the percentage of absorbance in Ctrl cells after subtraction of background absorbance. In addition, astrocytes were plated in matrigel-coated 48-well plates and exposed to 1 μM rotenone in N2 medium for 24 h. Subsequently, cells were fixed and stained for cleaved CASPASE-3 and the number of cleaved CASPASE-3 positive astrocytes was determined using Image J and the cell counter plugin.

**Neuronal differentiation**

NPCs were differentiated into neurons by incubation with N2B27 medium supplemented with 1 µM SAG (Cayman Chemical), 2 ng/ml BDNF (Peprotech), 2 ng/ml GDNF (Peprotech) and 100 µM AA for 6 days and afterwards with N2B27 medium supplemented with 2 ng/ml BDNF, 2 ng/ml GDNF, 0.5 ng/ml TGF-β3 (Peprotech), 100 µM dbcAMP (Sigma) and 100 µM AA. Additionally, 5 ng/ml Activin A (Peprotech) were added to the medium at days 7 through 9. After 9 days of neuronal differentiation, cells were detached, singularized by treatment with accutase and replated at densities of 2.2 x 10^5^ neurons in 12-well plates and 1.5 x 10^5^ neurons in 24-well plates containing glass coverslips. After 25 days of differentiation, cells were fixed in 4% PFA for immunocytochemical analyses or lysed in RLT buffer (Qiagen) for RNA preparation.

**Neuron and astrocyte co-cultures and FACS**

Co-culture experiments were conducted to assess a potential impact of N279K *MAPT* astrocytes on neurons. Ctrl-1 NPCs were labeled with GFP by lentiviral transduction with the LVTHM vector^6^ as previously described^3^. Labeled Ctrl-1 NPCs were subsequently differentiated into neurons applying the protocol described above. After 10 days of neuronal differentiation, cells were detached and singularized by treatment with acctuase. Neurons were added to FTD-1 , FTD-2 or Ctrl-2 astrocyte cultures at densities of 3.5 x 10^6^ neurons in a 10 cm dish and 1 x 10^5^ neurons per well in 24-well plates containing glass coverslips. Astrocytes had been plated 3 days before at day 31 of astrocyte differentiation at densities of 5 x 10^5^ cells in matrigel-coated 10 cm dishes and 1.5 x 10^4^ cells per well in matrigel-coated 24-well plates. Neurons were co-cultured with astrocytes in neuronal differentiation medium for 21 days. After that, cells were either fixed in 4% PFA or cells were detached and singularized by treatment with trypsin. Following washing with PBS, singularized cells were filtered through a 70 µm nylon mesh and sorted for GFP^+^ cells using a FACSAria cell sorter (BD Biosciences) to separate neurons from astrocytes. GFP^+^ neurons were collected in neuronal differentiation medium, before being pelletized and lysed in RLT-buffer. For whole genome expression analysis, total RNA was extracted using the RNeasy micro kit (Qiagen) and biotinylated cRNAs were generated using the TargetAmp 2-Round Biotin-aRNA Amplification Kit 3.0 (Epicenter). For qRT-PCR analysis, cRNA was reverse transcribed into cDNA using the High Capacity cDNA reverse Transcription Kit (Applied Biosystems) and 12 h of reverse transcription. cDNA was then used for qRT-PCR analysis.

**Quantification of neurons, neurite density, synaptogenesis and vulnerability to rotenone-induced stress**

To assess the influence of astrocytes on the vulnerability of neurons to rotenone-induced stress, co-cultured cells were incubated with neuronal differentiation medium lacking supplements but containing 400 nM rotenone (Sigma) for 48 h. Cells were subsequently fixed and stained for βIII-Tubulin and cleaved CASPASE-3. The number of neurons per visual field and the number of cleaved CASPASE-3-positive neurons was determined using Image J and the cell counter plugin. Additionally, the effect of astrocyte-conditioned medium on the vulnerability of neurons to rotenone-induced stress was determined. Astrocyte-conditioned medium was harvested from FTD-2 and Ctrl-2 astrocytes cultures after 2 days of conditioning. Neurons at day 12 of differentiation were cultured with either FTD-2 or Ctrl-2 conditioned media for 14 days during which the medium was refreshed every other day and neurons were subsequently stressed with 400 nM rotenone. Neurons were stained for βIII-Tubulin and cleaved CASPASE-3 and images were analyzed using Image J and the cell counter plugin.

Neurite density of neurons after co-culture with astrocytes was determined by staining of fixed cultures with an anti-GFP antibody. Images were taken with a Zeiss LSM700 confocal microscope and image analysis was performed using the ImageJ software and the color pixel plugin. The percentage of GFP positive signals over all signals was determined for 10 images per co-culture setup and per biological replicate. For quantification of synaptophysin expression in neurons after co-culture with astrocytes, fixed neurons were co-stained with anti-GFP and anti-Synaptophysin antibodies and Z-stack images were taken using a Zeiss LSM700 confocal microscope. Using the colocalization software from ZEN (Carl Zeiss Microimaging, LLC.), colocalization coefficients were determined to quantify the percentage of GFP-positive signals that were also positive for synaptophysin. A minimum of 3 single plane confocal images of a Z-stack were analyzed and the mean of the colocalization coefficients was calculated. 15 Z-stacks were analyzed per co-culture setup and per biological replicate.

**Table S1:** Primers used for qRT-PCR.

| *GAPDH*_for | CTG GTA AAG TGG ATA TTG TTG CCA T |
| --- | --- |
| *GAPDH*_rev | TGG AAT CAT ATT GGA ACA TGT AAA CC |
| *GFAP*_for | GGA AGA TTG AGT CGC TGG AG |
| *GFAP*_rev | ATA CTG CGT GCG GAT CTC TT |
| *S100β*_for | AAA GAG CAG GAG GTT GTG GA |
| *S100β*_rev | CGT GGC AGG CAG TAG TAA CC |
| *ALDH1L1*_for | TTG GGG TTT GTG GCA TCA TC |
| *ALDH1L1*_rev | AGC TCT GCA AAC TTC AAG GC |
| *SLC1A2*_for | CAT GGA TGG TAC AGC CCT TT |
| *SLC1A2*_rev | AGC AGG CTG ATG TCC TCT GT |
| *SLC1A3*_for | CTC ACA GTC ACC GCT GTC AT |
| *SLC1A3*_rev | CCA TCT TCC CTG ATG CCT TA |
| *MAPT_total*_for | CTC GCA TGG TCA GTA AAA GCA A |
| *MAPT_total*_rev | GGG TTT TTG CTG GAA TCC TGG T |
| *MAPT_Exon10*_for | CCA AGT GTG GCT CAA AGG AT |
| *MAPT_Exon12*_rev | CCC AAT CTT CGA CTG GAC TC |
| *ANXA2*_for | GGA CGC GAG ATA AGG TCC TG |
| *ANXA2*_rev | GCT TTC TGG TAG TCG CCC TT |
| *GAS7*_for | ATC AAG AAG GCG CGG AGA AA |
| *GAS7*_rev | GCT GCC GGA TCA TCT CTA CC |
| *NPY*_for | CGG AGG ACA TGG CCA GAT AC |
| *NPY*_rev | TCA AGC CGA GTT CTG GGA AC |
| *TCEAL7*_for | GAA CGC CAG CAA ACA GAA GG |
| *TCEAL7*_rev | GGT CCC GAG AAT GCC TAT GG |
| *MMP14*_for | GGT GCC CTA TGC CTA CAT CC |
| *MMP14*_rev | CAC AGC CAC CAG GAA GAT GT |
| *CXCL12*_for | GAA AGC CAT GTT GCC AGA GC |
| *CXCL12*_rev | TCG GGT CAA TGC ACA CTT GT |
| *DYSF*_for | TTT GCG GGG AAA ATG CTG TG |
| *DYSF*_rev | ATG GAG GGA AAC ATG GCA GG |
| *EN1*_for | GCA ACC CGG CTA TCC TAC TT |
| *EN1*_rev | CGC TTG TCC TCC TTC TCG TT |
| *MAOB*_for | CTC CCC TTG CAG AAG AGT GG |
| *MAOB*_rev | CCA CAG GAA CCA GAG AGC AG |
| *NELL2*_for | CTT GTC ATG CCC CAG GGA TT |
| *NELL2*_rev | ACA GCC GTC TAT CCA GGA CT |
| *RANBP17_*for | ATC TTC GAG GGA TTG CCT TT |
| *RANBP17_*rev | AAT TCA AAC GCT GGG ATC TG |
| *TERF1_for* | ATG GAA CCC AGC AAC AAG AC |
| *TERF1_rev* | CCT CAC GCC AGA TCT CAA AT |
| *TERF2_for* | TAC CCA AAG GCA AGT GGA AC |
| *TERF2_rev* | TCC CCA TAT TTC TGC ACT CC |
| *SIRT1_for* | CTG GAC AAT TCC AGC CAT CT |
| *SIRT1_rev* | GCA CCT AGG ACA TCG AGG AA |
| *SIRT6_for* | CCA AGT TCG ACA CCA CCT TT |
| *SIRT6_rev* | CGG ACG TAC TGC GTC TTA CA |
| *CDKN1C_*for | CAG GAG CCT CTC GCT GAC |
| *CDKN1C_*rev | CTT CTC AGG CGC TGA TCT CT |
| *CXCR4_for* | ATC AGT CTG GAC CGC TAC CT |
| *CXCR4_rev* | ATC TGC CTC ACT GAC GTT GG |

**Supplementary References**

^1^ Ehrlich, M. *et al.* Distinct Neurodegenerative Changes in an Induced Pluripotent Stem Cell Model of Frontotemporal Dementia Linked to Mutant TAU Protein. *Stem Cell Reports* **5,** 83-96 (2015).

^2^ Reinhardt, P. *et al.* Derivation and expansion using only small molecules of human neural progenitors for neurodegenerative disease modeling. *PLoS One* **8,** e59252 (2013).

^3^ Hargus, G. *et al.* Origin-dependent neural cell identities in differentiated human iPSCs in vitro and after transplantation into the mouse brain. *Cell Rep* **8,** 1697-1703 (2014).

^4^ Irizarry, R. A. *et al.* Summaries of Affymetrix GeneChip probe level data. *Nucleic Acids Res* **31,** e15 (2003).

^5^ Kim, J. B. *et al.* Direct reprogramming of human neural stem cells by OCT4. *Nature* **461,** 649-653 (2009).

^6^ Wiznerowicz, M. & Trono, D., Conditional suppression of cellular genes: lentivirus vector-mediated drug-inducible RNA interference. *J Virol* **77,** 8957-8961 (2003).

**Supplementary Figures**

**
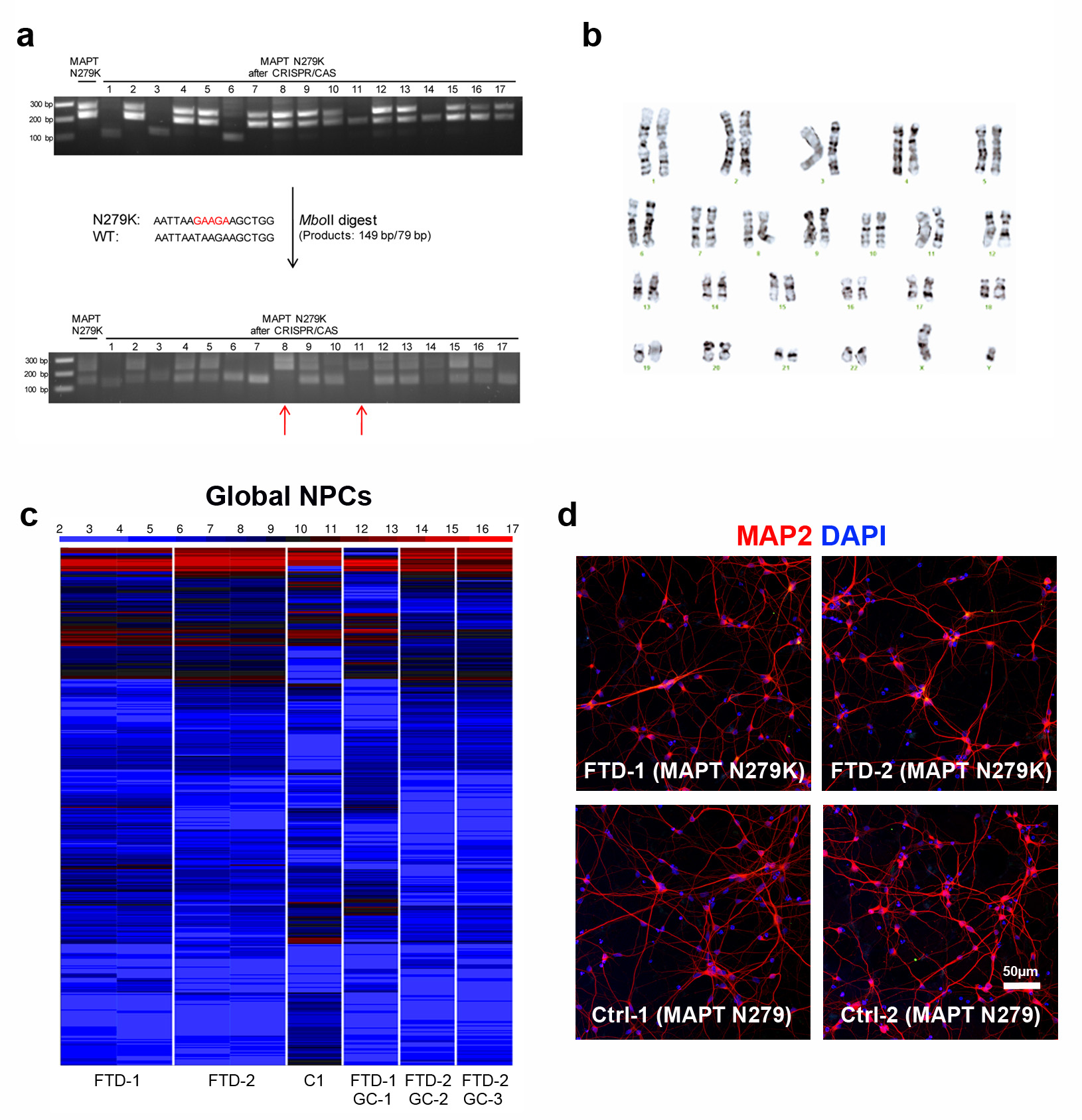
**

**Supplementary Figure S1: Screening for and characterization of CRISPR/Cas9 gene-corrected neural progenitor cells (NPCs).**

**(a)** Genomic DNA was isolated from puromycin-resistant CRISPR/Cas9/ssODN transfected FTD N279K *MAPT* NPCs. A 228 bp fragment spanning the mutation-containing region in exon 10 of *MAPT* was amplified via PCR and was digested with *Mbo*II. *Mbo*II does not cut the rescued wildtype N279 *MAPT* sequence in indicated clones (red arrows). **(b)** G-banded karyotype analysis of isogenic Ctrl NPCs (FTD-1 GC-1). **(c)** Heat map of transcriptome profiles in FTD and Ctrl NPCs. **(d)** Immunostaining of FTD and Ctrl neurons for MAP2 (red). Nuclei were counterstained with DAPI (blue). Scale bar = 50 µm.


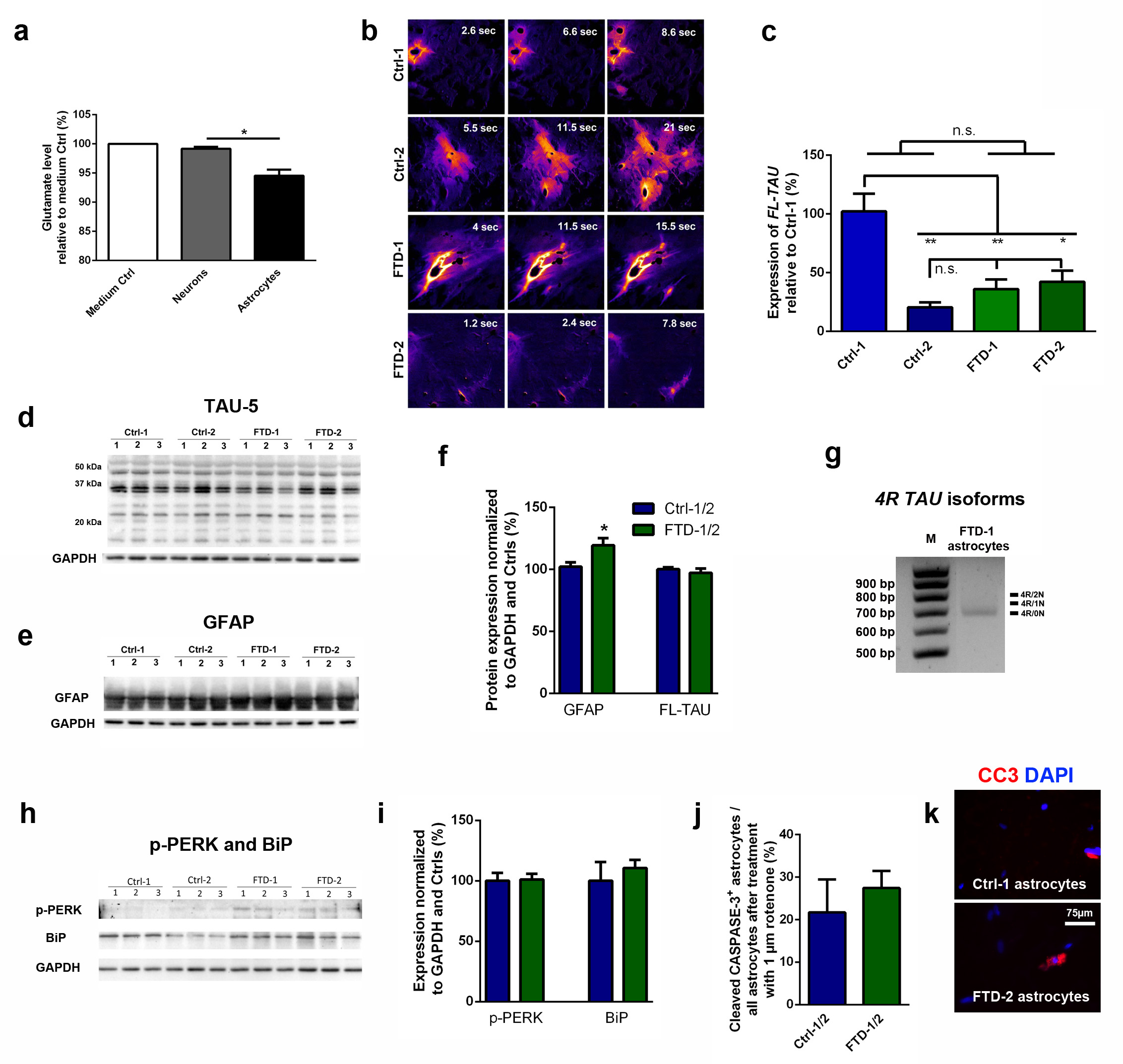


**Supplementary Figure S2: Characterization of astrocytes from Ctrl and FTD NPCs.**

**(a)** Quantification of glutamate uptake in Ctrl-1 astrocytes and Ctrl-1 neurons. Note the lack of glutamate uptake in neurons. Data are represented as mean of replicates from three independent differentiation experiments (n=3 per group) + SEM. **(b)** Fluorescence analysis of FTD and Ctrl astrocytes demonstrating propagation of calcium waves over time. **(c)** Quantification of full-length *TAU* (*FL-TAU*) expression in Ctrl-1, Ctrl-2, FTD-1 and FTD-2 astrocytes via qRT-PCR. Note increased expression in the non-isogenic Ctrl line (Ctrl-1) but no significant difference between lines Ctrl-2, FTD-1 and FTD-2. Data are represented as mean of replicates from three independent differentiation experiments (n=3 per line) + SEM. One-way ANOVA with post hoc Bonferroni test was performed for statistical analysis (^*^*p*<0.05, ^**^*p*<0.01). **(d-e)** Western blot expression analysis of (d) TAU (clone TAU-5) and (e) GFAP protein in FTD and Ctrl astrocytes. GAPDH was used as loading control. Independent replicates are shown for each line. **(f)** Quantification of TAU and GFAP protein expression in FTD and Ctrl astrocytes. **(g)** qRT-PCR expression analysis of *4R-TAU* isoforms using primers binding in exon 1 and exon 10 of *MAPT*. NPC-derived astrocytes express the shortest *4R-TAU* isoform (*4R/0N*, 676 bp), while other *4R-TAU* isoforms (*4R/1N*, 764 bp; *4R/2N*, 851 bp) are undetected. **(h)** Western blot expression analysis of p-PERK and BiP protein in FTD and Ctrl astrocytes. GAPDH was used as loading control. Independent replicates are shown for each line. **(i)** Quantification of p-PERK and BiP protein expression in FTD and Ctrl astrocytes. **(j)** Quantification of cleaved CASPASE-3-positive astrocytes 48 h after application of 1 µm rotenone. **(k)** Immunostainings of FTD and Ctrl astrocytes for cleaved CASPASE-3 (CC3, red). Nuclei were counterstained with DAPI (blue). Scale bar = 75 µm. Data in panels f, i and j are represented as mean of replicates from three independent experiments per line + SEM (n=3 per line; n=6 per group). Student’s t-test was performed for statistical analysis (^*^*p*<0.05).

**
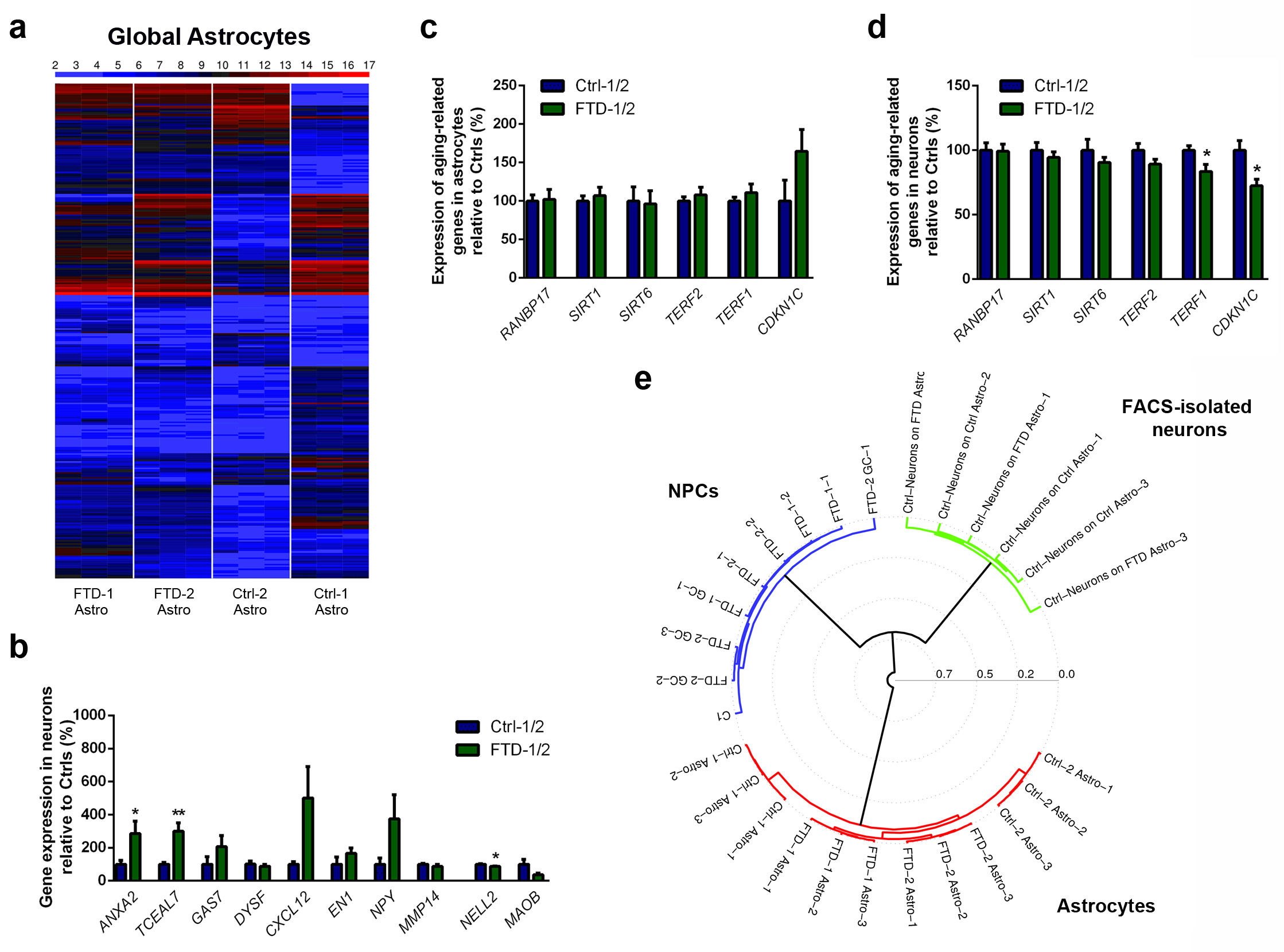
**

**Supplementary Figure S3: Transcriptome profiles in FTD and Ctrl astrocytes, expression analysis in Ctrl and FTD neurons and expression of aging-associated genes in FTD and Ctrl astrocytes.**

**(a)** Heat map of whole genome expression profiles in FTD and Ctrl astrocytes. **(b)** qRT-PCR expression analyses in FTD and Ctrl neurons of genes differentially expressed in FTD and Ctrl astrocytes. **(c-d)** qRT-PCR expression analysis of aging-associated genes in (c) FTD and Ctrl astrocytes and in (d) FTD and Ctrl neurons. Data in panels b-d are represented as mean of replicates from three independent differentiation experiments (n=3 per line; n=6 per group) + SEM. Student’s t-test was performed for statistical analysis (^*^*p*<0.05; ^**^*p*<0.01). **(e)** Hierarchical cluster analysis of 255 genes, which are upregulated in the aging brain. Note that the FTD astrocytes form one cluster and separate from Ctrl-1 and Ctrl-2 astrocytes.

**
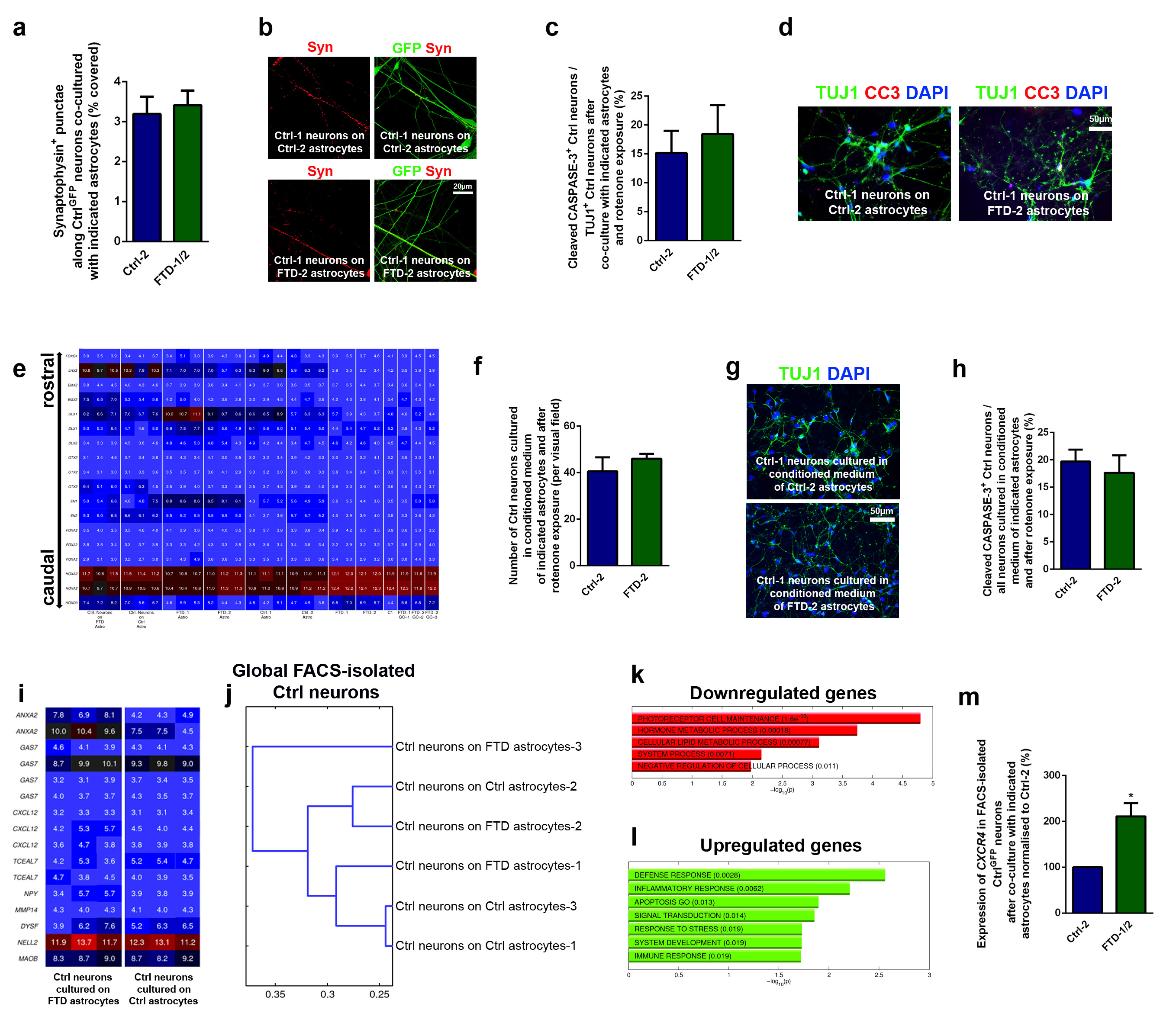
**

**Supplementary Figure S4: Co-culture of control neurons with FTD astrocytes results in changes in gene expression profiles in previously healthy neurons.**

**(a)** Quantification of the coverage of Ctrl^GFP^ neurons with synaptophysin-positive punctae. **(b)** Immunostainings of Ctrl^GFP^ neurons cultured on either FTD or Ctrl astrocytes for synaptophysin (Syn; red). Nuclei were counterstained with DAPI (blue). Scale bar = 20 µm. **(c)** Quantification of the percentage of cleaved CASPASE-3-positive neurons after co-culture with either FTD or Ctrl astrocytes and rotenone treatment. **(d)** Immunostainings of Ctrl neurons cultured on either FTD or Ctrl astrocytes for cleaved CASPASE-3 (CC3; red) and βIII-Tubulin (TUJ1; green). Nuclei were counterstained with DAPI (blue). Scale bar = 50 µm. **(e)** Heat map demonstrating the expression of regional genes along the rostro-caudal axis in FTD and Ctrl NPCs, FTD and Ctrl astrocytes and in Ctrl neurons co-cultured with either FTD or Ctrl astrocytes. **(f)** Quantification of Ctrl neurons cultured in conditioned medium from either FTD-2 or Ctrl-2 astrocytes and after rotenone treatment. **(g)** Immunostaining of Ctrl neurons cultured in conditioned medium from either FTD-2 or Ctrl-2 astrocytes and after rotenone treatment for TUJ1 (green). Nuclei were counterstained with DAPI (blue). Scale bar = 50 µm. **(h)** Quantification of the percentage of cleaved CASPASE-3-positive Ctrl neurons cultured in conditioned medium form either FTD-2 or Ctrl-2 astrocytes and after rotenone treatment. **(i)** Heat map showing the expression of genes differentially regulated in FTD astrocytes in FACS-isolated Ctrl^GFP^ neurons after co-culture with either FTD or Ctrl astrocytes. **(j)** Hierarchical cluster dendrogram of global expression profiles from Ctrl^GFP^ neurons co-cultured with either FTD or Ctrl astrocytes. **(k-l)** Functional annotation of (k) downregulated and (l) upregulated genes in Ctrl^GFP^ neurons co-cultured with FTD astrocytes with p-values. **(m)** qRT-PCR expression analysis of *CXCR4* in FACS-isolated Ctrl^GFP^ neurons after co-culture with either FTD or Ctrl astrocytes. Data in panels a, c, f, h and m are represented as mean of replicates from three independent co-culture experiments (n=3 per line) + SEM. Student’s t-test was performed for statistical analysis (^*^*p*<0.05).

**
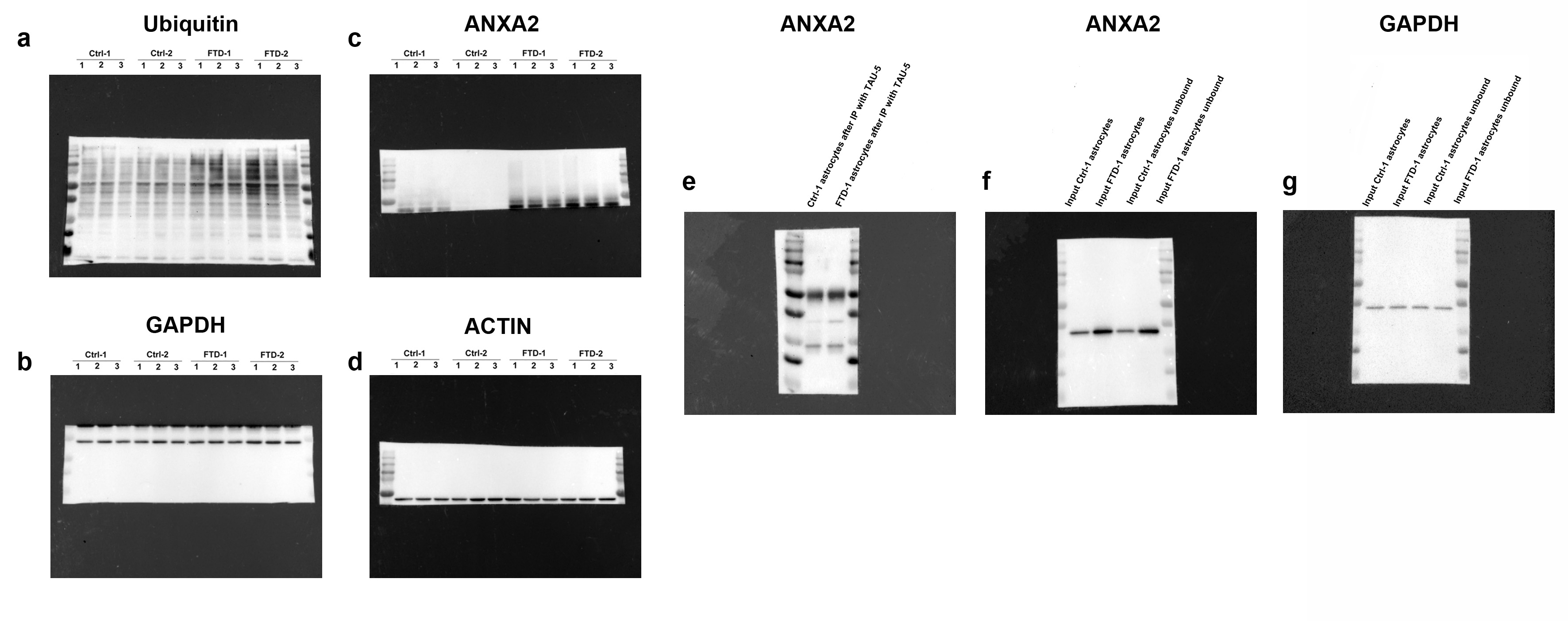
**

**Supplementary Figure S5: Full-size western blots showing expression of ubiquitin, ANXA2, beta-ACTIN and GAPDH in FTD and Ctrl astrocytes.**

**(a)** Uncropped western blot showing expression of ubiquitin in human NPC-derived FTD and Ctrl astrocytes. **(b)** Uncropped western blot showing expression of GAPDH in human NPC-derived FTD and Ctrl astrocytes. Images in (a) and (b) correspond to images in panel k of Fig. 2. **(c)** Uncropped western blot showing expression of ANXA2 in FTD and Ctrl astrocytes. **(d)** Uncropped western blot showing expression of β-ACTIN (ACTIN) in FTD and Ctrl astrocytes. Images in (c) and (d) correspond to images in panel c of Fig. 3. **(e)** Uncropped western blot showing ANXA2 protein in Ctrl-1 and FTD-1 astrocytes after co-immunoprecipitation using TAU-5 antibody for extraction from protein lysates. TAU-5 immunoprecipitates were analyzed by western blot and probed by ANXA2 antibody. **(f)** Uncropped western blot showing ANXA2 expression in input fractions from Ctrl-1 and FTD-1 astrocytes. **(g)** Uncropped western blot showing GAPDH expression in input fractions from Ctrl-1 and FTD-1 astrocytes. Images in (e), (f) and (g) correspond to images in panel e of Fig. 3.


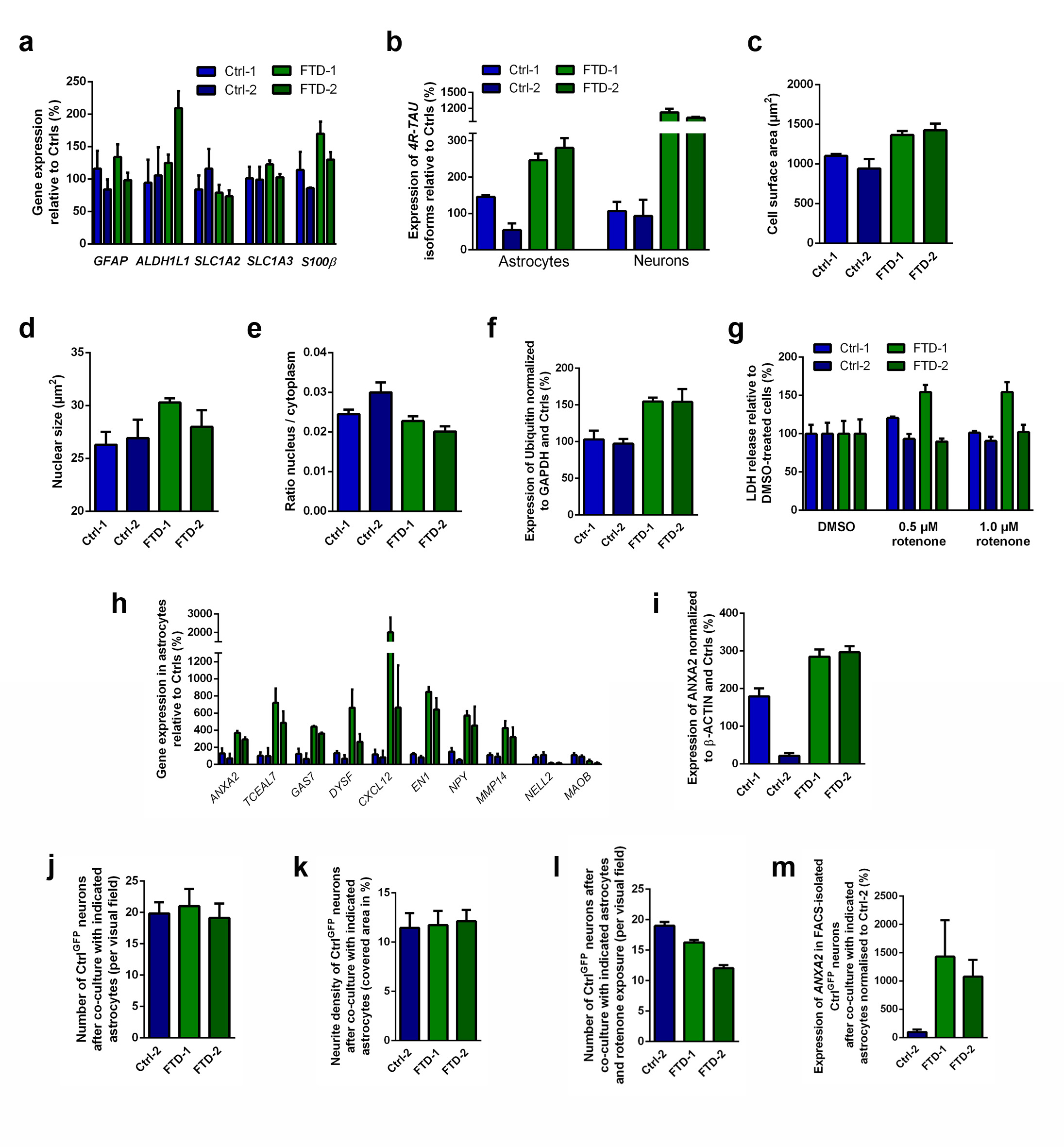


**Supplementary Figure S6: Presentation of bar diagrams depicting non-pooled data of FTD and Ctrl lines**

**(a-b)** qRT- PCR expression analysis of astrocyte marker genes in FTD and Ctrl astrocytes (a) and of *4R-TAU* isoforms in FTD and Ctrl astrocytes and neurons (b). **(c-e)** Quantification of the cell size (c), nuclear size (d) and nucleus / cytoplasm ratio (e) of FTD and Ctrl astrocytes. **(f)** Quantification of ubiquitin protein expression in FTD and Ctrl astrocytes. **(g)** Effect of oxidative stress on FTD and Ctrl astrocyte viability as analyzed by measurement of LDH release after 48 h of rotenone treatment. Data are represented as mean of four replicates per line (n=4 per line) + SEM. **(h)** qRT-PCR expression analysis of differentially expressed genes in FTD and Ctrl astrocytes. **(i)** Quantification of ANXA2 protein expression in FTD and Ctrl astrocytes. Data in panels a-f and h-i are represented as mean of replicates from three independent differentiation experiments per line (n=3 per line) + SEM. **(j-k)** Quantification of the number of Ctrl^GFP^ neurons (j) and their neurite density (k) after co-culture with either FTD or Ctrl astrocytes. **(l)** Effect of oxidative stress on Ctrl^GFP^ neurons after co-culture with either FTD or Ctrl astrocytes as analyzed by the number of surviving neurons after 48 h of rotenone treatment. **(m)** qRT-PCR expression analysis of ANXA2 in FACS-isolated Ctrl^GFP^ neurons after co-culture with either FTD or Ctrl astrocytes. Data in panels j-m are represented as mean of three replicates per line from three independent co-culture experiments (n=3 per line).
